# Assessment of malnutrition in cancer patients: a geriatric approach with the mini nutritional assessment

**DOI:** 10.3389/fnut.2025.1590137

**Published:** 2025-05-09

**Authors:** Ozlem Dogan, Hayriye Sahinli, Dogan Yazilitas

**Affiliations:** ^1^Adiyaman University Training and Research Hospital, Department of Medical Oncology, Adıyaman, Türkiye; ^2^Ankara Etlik City Hospital, Department of Medical Oncology, Ankara, Türkiye

**Keywords:** malnutrition, geriatrics, depression, mini nutritional assessment, cancer

## Abstract

**Background and objectives:**

Malnutrition is a common problem among cancer patients, significantly impacting clinical outcomes and quality of life. This study aimed to evaluate the prevalence of malnutrition and its associated factors in geriatric cancer patients undergoing chemotherapy.

**Materials and methods:**

This prospective study included 471 patients aged 65 years and older, conducted at Ankara Etlik City Hospital between January and December 2023. Patients’ demographic, clinical, and nutritional statuses were assessed using the Mini Nutritional Assessment (MNA). Nutritional status was classified as normal (MNA ≥ 24), at risk of malnutrition (MNA 17–23.5), and malnourished (MNA < 17). Depression and insomnia were evaluated using the Geriatric Depression Scale (GDS) and the Insomnia Severity Index (ISI), respectively. Factors associated with malnutrition were analyzed statistically.

**Results:**

Malnutrition was identified in 20.5% of the patients. Malnutrition was significantly associated with radiotherapy (*p* = 0.001), surgical history (*p* = 0.001), adjuvant therapy (*p* = 0.002), metastatic disease (*p* = 0.011), low BMI (*p* < 0.001), high depression scores (*p* < 0.001), moderate-to-severe insomnia (*p* < 0.001), and the presence of comorbidities (*p* = 0.022). However, no significant association was found between pain and malnutrition (*p* = 0.07).

**Conclusion:**

This study highlights the multifactorial nature of malnutrition in geriatric cancer patients and emphasizes the importance of regular nutritional assessments using validated tools like MNA. Early detection and intervention can improve clinical outcomes and quality of life. However, the study has certain limitations, including being single-center, the use of self-reported measures, and the exclusion of palliative patients, which may affect the generalizability of the results.

## Introduction

Malnutrition, defined as inadequate nutrient intake or absorption, is a critical health issue linked to adverse clinical outcomes (1). It is prevalent among cancer patients, driven by tumor-related metabolic changes, insufficient intake, and treatment-related gastrointestinal side effects like mucositis, diarrhea, and nausea (2). Studies have reported that the prevalence of malnutrition in cancer patients ranges from 20 to 70%, with severe consequences on clinical outcomes and quality of life (1–4). Early detection of malnutrition risk in cancer patients is crucial to improving outcomes and enhancing their quality of life.

Medical and technological advancements have contributed to the significant growth of the global elderly population. Consequently, approximately 60% of cancer patients are older adults (5). In this population, malnutrition is further exacerbated by cancer-related factors, resulting in prolonged hospital stays, poorer treatment outcomes, decreased survival rates, and significant declines in quality of life (2). Given these factors, nutritional assessment becomes an essential component of care in this vulnerable population. Timely and effective interventions can significantly improve their nutritional status.

The Mini Nutritional Assessment (MNA) is a validated tool recommended by ESPEN for evaluating malnutrition in cancer patients (6). Specifically designed for the elderly, the MNA includes 18 questions covering dietary intake and anthropometric measurements, with a total score of 30 points. Nutritional status is categorized as normal (24–30), at risk of malnutrition (17–23.5), or malnourished (<17) (7).

This study aimed to utilize the Mini Nutritional Assessment to investigate malnutrition prevalence, identify patients at risk, and explore associated factors among cancer patients aged 65 years and older undergoing treatment.

## Materials and methods

A prospective study was conducted at Ankara Etlik City Hospital between January and December 2023. A total of 471 patients aged 65 years and older, who were receiving chemotherapy in the outpatient treatment unit and provided written informed consent to participate, were included in the study. Patients who were receiving only oral therapy, under palliative care, or lacked sufficient cognitive function to answer survey questions were excluded from the study.

The demographic and clinicopathological characteristics of the patients were obtained from the hospital’s electronic medical record system and patient files. Collected data included age, gender, ECOG performance status, BMI, comorbidities, alcohol use, education and occupational history, cancer type and stage, history of radiotherapy or surgery, knowledge about the disease, and the duration since cancer treatment initiation. To evaluate the level of social support, patients were categorized based on their living arrangements as either having strong social support (living with family members) or weak social support (living alone, with a caregiver, or in a nursing home). The study was conducted in accordance with the principles of the Declaration of Helsinki and was approved by the Ethics Committee of Health Sciences University Diskapi Yildirim Beyazit Training and Research Hospital.

### Assessment of malnutrition

The Mini Nutritional Assessment (MNA) is a validated tool developed specifically to assess malnutrition in elderly patients. It has been translated into multiple languages and consists of simple anthropometric measurements and short questions. The anthropometric measurements include mid-arm circumference, calf circumference, and body mass index (BMI). Other questions cover dietary intake (e.g., number of meals consumed, food and fluid intake), general assessment (e.g., lifestyle, number of medications, presence of stress), and self-assessment (e.g., perception of health and nutrition) (7). Although the Global Leadership Initiative on Malnutrition (GLIM) criteria are widely used for nutritional evaluation, the MNA was selected for this study due to its specific validation in geriatric populations and its practicality in routine outpatient settings.

During chemotherapy, patients completed the questionnaire either independently or with the assistance of relatives or healthcare personnel. Anthropometric measurements were taken and recorded by the same healthcare personnel using standardized equipment. BMI was calculated by dividing weight (in kilograms) by height squared (in meters squared). Mid-arm circumference was measured at the midpoint between the lateral projection of the scapula’s acromion and the lower edge of the olecranon process of the ulna, while calf circumference was measured at the widest part of the calf (8).

The MNA consists of 18 questions and has a total score of 30 points. Nutritional status was classified into three categories based on the score: normal nutritional status (24–30), at risk of malnutrition (17–23.5), and malnourished (<17).

### Assessment of depression

The 15-item short form of the Yesavage Geriatric Depression Scale (GDS), validated and adapted for use in Turkey, was applied to all patients. This tool is specifically designed for geriatric populations, with responses recorded as “yes” or “no.” Scores were categorized as follows: 0–4 indicated a low level of depressive symptoms, while scores of 5 or higher were classified as a high level of depressive symptoms (9).

### Assessment of insomnia

The Insomnia Severity Index (ISI) was employed to evaluate the severity of insomnia. This tool consists of seven items, with responses rated on a scale ranging from 0 to 4. Based on the total score, insomnia was categorized as follows: 0–7 (no clinically significant insomnia), 8–14 (mild insomnia), 15–21 (moderate insomnia), and 22–28 (severe insomnia) (10).

### Statistical analysis

All statistical analyses were performed using SPSS version 21.0 (SPSS Inc., Chicago, IL). The normality of continuous variables was assessed using the Kolmogorov–Smirnov and Shapiro–Wilk tests, along with the evaluation of skewness and kurtosis values, given the known sensitivity of the Kolmogorov–Smirnov test in large sample sizes. Non-normally distributed continuous variables were expressed as medians (min–max). The relationships between clinicopathological characteristics and nutritional status were evaluated using the Chi-square (X^2^) test and Fisher’s Exact test, with Bonferroni correction applied for multiple comparisons where appropriate. A *p*-value of <0.05 was considered statistically significant.

## Results

A total of 471 patients were included in the study. Of these, 282 (59.9%) were male, and 189 (40.1%) were female. In terms of age distribution, 362 patients (76.9%) were between 65 and 74 years old, while 109 patients (23.2%) were aged 75 and older. Regarding performance status (PS), 369 patients (78.3%) had a PS score of 1, and 102 patients (21.7%) had a PS score of 0. In terms of comorbidities, 267 patients (56.7%) had no comorbid conditions, while 204 patients (43.3%) had one or more comorbidities. A majority of the patients, 437 (92.8%), were not working, while 34 (7.2%) were employed. When evaluating social support, 432 patients (91.7%) were categorized as having strong social support, while 39 patients (8.3%) were categorized as having weak social support.

In terms of cancer stage, 237 patients (50.3%) were in the localized stage, and 234 patients (49.7%) were in the metastatic stage. Regarding alcohol use, 438 patients (93%) reported no alcohol consumption. Additionally, 444 patients (94.3%) were knowledgeable about their disease. Among the included patients, 464 (98.5%) were not using antidepressant medications. In terms of adjuvant therapy, 260 patients (55.2%) had not received adjuvant therapy. The number of patients with no history of radiotherapy was 354 (75.2%). Finally, when examining the time elapsed since the start of treatment, 267 patients (56.7%) were found to have been under treatment for less than 6 months.

Malnutrition was identified in 97 patients (20.5%). The prevalence of malnutrition was significantly higher among patients who had undergone radiotherapy (*p* = 0.001), had a history of surgery (*p* = 0.001), received adjuvant therapy (*p* = 0.002), or were in the metastatic stage (*p* = 0.011). Malnutrition was also significantly more common in patients with high depression levels (*p* < 0.001), low BMI (*p* < 0.001), mild-to-severe insomnia (*p* < 0.001), and those with one or more comorbidities (*p* = 0.022). However, no statistically significant association was found between pain and malnutrition (*p* = 0.07) (Table 1). These results highlight the multifactorial nature of malnutrition and suggest that clinical, psychological, and treatment-related factors should be routinely assessed in elderly cancer patients.

**Table 1:** Relationship between patient characteristics and nutritional status.

Characteristics	Total *n* (%)	Malnutrition *n* (%)	Normal nutrition *n* (%)	*p* value
Gender				
Female	189 (40.1)	34 (35.1)	155 (41.4)	0.252
Male	282 (59.9)	63 (64.9)	219 (58.6)	
Age group				
65–74	362 (76.9)	73 (75.3)	289 (77.3)	0.675
>75	109 (23.1)	24 (24.7)	85 (22.79)	
ECOG				
0	102 (21.7)	24 (24.7)	78 (20.9)	
1	369 (78.3)	73 (75.3)	296 (79.1)	0.408
Comorbidities				
None	267 (56.7)	45 (46.7)	222 (59.4)	**0.022**
≥1	204 (43.3)	52 (53.6)	152 (40.6)	
Employment status				
Yes	34 (7.2)	3 (3.1)	31 (8.3)	0.082
No	437 (92.8)	94 (96.9)	343 (91.7)	
Social support				
Weak	39 (8.3)	12 (12.4)	27 (7.2)	0.101
Strong	432 (91.7)	85 (87.6)	347 (92.8)	
Stage				
01-03	237 (50.3)	60 (61.9)	177 (47.3)	**0.011**
4	234 (49.7)	37 (38.1)	197 (52.7)	
Alcohol use				
Yes	33 (7)	8 (8.2)	25 (6.7)	0.591
No	438 (93)	89 (91.8)	349 (93.3)	
Aware of disease				
Yes	444 (94.3)	88 (90.7)	356 (95.2)	0.092
No	27 (5.7)	9 (9.3)	18 (4.8)	
Use of antidepressant				
Yes	7 (1.5)	0 (0)	7 (1.9)	0.354
No	464 (98.5)	97 (100)	367 (9.8)	
Adjuvant therapy				
Evet	211 (44.8)	57 (58.8)	154 (41.2)	**0.002**
Hayır	260 (55.2)	40 (41.2)	220 (58.8)	
Radiotherapy				
Yes	117 (24.8)	12 (12.4)	105 (28.1)	**0.001**
No	354 (75.2)	85 (87.6)	269 (71.9)	
Surgical history				
Yes	208 (44.2)	58 (59.8)	150 (40.1)	**0.001**
No	263 (55.8)	39 (40.2)	224 (59.9)	
Time since cancer treatment				
<6 ay	267 (56.7)	58 (59.8)	209 (55.9)	0.488
≥6 ay	204 (43.3)	39 (40.2)	165 (44.1)	
Depression scale				
0–4	280 (59.4)	32 (33)	248 (66.3)	**<0.001**
≥5	191 (40.6)	65 (67)	126 (33.7)	
Insomnia				
0–7	291 (61.7)	43 (14.8)	248 (85.2)	**<0.001**
08-28	180 (38.3)	54 (30.0)	126 (70.0)	
Body mass index				
<18.5	31 (6.6)	21 (21.6)	10 (2.7)	**<0.001**
18.5–25	244 (52)	59 (60.8)	185 (49.7)	
≥25	194 (41.4)	17 (17.5)	177 (47.6)	
Cancer type				
Gastrointestinal	208 (44.2)	49 (50.5)	159 (42.5)	0.157
Other	263 (55.8)	48 (49.5)	215 (57.5)	
Pain				
(Median, min-max)	1 (0–9)	1 (0–7)	1 (0–9)	0.07

Bold values indicate the *p*-value of <0.05 was considered statistically significant.

## Discussion

This study aimed to evaluate the relationship between malnutrition and various clinical and demographic characteristics in cancer patients. Malnutrition was identified in 20.5% of the patients, which aligns with the prevalence rates reported in the literature, ranging from 20 to 70%. Similarly, Arends et al. (1) highlighted this wide range of malnutrition prevalence in their 2017 review, which included recommendations from the European Society for Clinical Nutrition and Metabolism (ESPEN). These findings underscore the prevalence of malnutrition in cancer patients and emphasize the need for regular nutritional assessments.

In agreement with the review published by Bossi et al. (11), our study demonstrated that malnutrition rates increase after radiotherapy and surgical interventions. Likewise, a study by Cao et al. (12) on esophageal cancer patients found that radiotherapy was associated with a higher risk of malnutrition. In our study, a significant relationship was observed between radiotherapy and malnutrition (*p* = 0.001), potentially due to side effects such as mucositis, xerostomia, and dysphagia, which can adversely affect nutritional status. Similarly, patients with a history of surgery had significantly higher rates of malnutrition (*p* = 0.001), which may be attributed to increased metabolic demands and the challenges of postoperative recovery.

In line with the findings of Firouzabadi et al. (13), our study showed that malnutrition rates were higher depending on the type of treatment and the stage of the disease. Patients who had undergone adjuvant therapy had significantly higher malnutrition rates (*p* = 0.002), likely due to treatment-related toxicities and appetite loss. A significant association was also found between metastatic disease and malnutrition (*p* = 0.011), supporting literature findings that advanced cancer stages can lead to an increased catabolic state and reduced dietary intake.

A strong relationship between malnutrition, depression, and insomnia was observed (*p* < 0.001). Hu et al. (14) reported that malnutrition increases the risk of depression in elderly patients. Similarly, O’Keeffe et al. (15) found that psychological distress, anxiety, and loneliness significantly contribute to malnutrition risk. Our study supports the bidirectional relationship between depression, insomnia, and malnutrition; while malnutrition exacerbates these conditions, depression and insomnia also negatively affect nutritional status. However, it is important to note that this relationship may be influenced by unmeasured confounding factors such as cancer type, disease stage, treatment-related toxicities, or socioeconomic status, which were not fully accounted for in our analysis.

Baracos et al. (16) concluded that chronic pain triggers inflammation, increasing catabolic processes and contributing to malnutrition. However, in our study, no statistically significant relationship was found between pain and malnutrition (*p* = 0.07). This discrepancy may be explained by differences in the patient population, the lack of detailed evaluation of pain’s source and severity, or other factors (e.g., BMI, depression, comorbidities) masking the effect of pain. Additionally, the absence of data on analgesic use and cancer type may have further confounded this relationship. Pain is also a highly subjective experience, and variations in patient perception, reporting, or underreporting may have limited the accuracy of our findings.

According to the Global Leadership Initiative on Malnutrition (GLIM) report published in 2019, low BMI is one of the key criteria for identifying malnutrition (17). Similarly, our study found a significant relationship between low BMI and malnutrition (*p* < 0.001). Malnutrition was more prevalent among patients with a BMI below 18.5, whereas those with a BMI above 25 had the lowest rates of malnutrition. Additionally, comorbidities were significantly associated with malnutrition (*p* = 0.022), likely due to their cumulative impact on overall health and functional status.

This study has several limitations. First, as a single-center study, the generalizability of the findings is limited. Conducting studies in diverse geographical locations and patient populations would enhance the applicability of the results. Second, although this study was prospectively designed, parameters such as depression and insomnia were assessed using self-reported questionnaires, increasing the risk of bias in the data. Moreover, the lack of detailed information on pain’s duration, severity, and source limited the comprehensive interpretation of results. Another limitation is that only patients receiving chemotherapy in the outpatient treatment unit were included, while those under palliative care or receiving oral therapy were excluded. Another limitation is potential selection bias, as patients with impaired cognitive function or those receiving palliative or oral therapy were excluded from the study. This may have resulted in underrepresentation of more vulnerable individuals and limits the generalizability of the findings. Finally, nutritional status was assessed solely using the MNA. Incorporating additional nutritional assessment tools could provide a more comprehensive evaluation.

## Conclusion

This study highlights the multifactorial nature of malnutrition in cancer patients and underscores the importance of regular nutritional assessments using validated tools such as the MNA. Factors such as treatment-related side effects, depression, insomnia, and low BMI were identified as significant contributors to malnutrition risk. These findings emphasize the need for multidisciplinary approaches and early intervention strategies to improve the nutritional status and overall outcomes of geriatric cancer patients. In clinical practice, routine screening for malnutrition should be integrated into standard oncologic care to optimize treatment tolerance and quality of life. Future studies should include multi-center, longitudinal designs and evaluate additional variables such as pain characteristics, socioeconomic factors, and functional status to provide a more comprehensive understanding of malnutrition in this vulnerable population.

## Data Availability

The raw data supporting the conclusions of this article will be made available by the authors, without undue reservation.
